# Prevalence and predictors of anaemia among adolescents in Bihar and Uttar Pradesh, India

**DOI:** 10.1038/s41598-022-12258-6

**Published:** 2022-05-17

**Authors:** Shekhar Chauhan, Pradeep Kumar, Strong P. Marbaniang, Shobhit Srivastava, Ratna Patel

**Affiliations:** 1 Department of Family & Generations, International Institute for Population Sciences, Mumbai, India; 2 Department of Survey Research & Data Analytics, International Institute for Population Sciences, Mumbai, India; 3 Department of Public Health and Mortality Studies, International Institute for Population Sciences, Mumbai, India; 4 Department of Statistics, Sankardev College, Shillong, India

**Keywords:** Public health, Health policy

## Abstract

In adolescents, anaemia has been linked to affecting physical disorders, growth, and mental retardation and also increases reproductive morbidities among adolescent girls during their womanhood. It is believed that with increasing age, females are more prone to anaemia than their male counterparts. Unfortunately, the anaemia intervention program, such as the National Nutrition Anaemia Prophylaxis Programme, primarily targets infants, young children, pregnant and lactation women, and not adolescents. Therefore, this study tries to fill this gap and study the prevalence of anaemia and the associated factors among adolescent boys and girls residing in Uttar Pradesh and Bihar, India. Secondary data analysis was performed on cross-sectional survey data from the Understanding the Lives of Adolescents and Young Adults survey. The sample size was 20,594 adolescents aged 10–19 years in Uttar Pradesh and Bihar, India. The outcome variable was anaemia, and the explanatory variables were age, education, working status, media exposure, marital status, received IFA and deworming tablets, BMI status, stunting status, wealth index, caste, religion, residence, and States. Descriptive statistics and bivariate analysis were used to find the preliminary results. Multinomial regression analysis was carried out to provide the adjusted estimates. Overall, anaemia was more prevalent among adolescent girls than adolescent boys (20% vs. 8.7%). Moderate/severe anaemia was 0.24 and 0.49 times less likely among adolescent boys and girls, respectively, who had 10 and above years of schooling than adolescents with no schooling (p < 0.01). Rural adolescent boys were 1.49 times (p < 0.05) more likely to suffer from moderate/severe anaemia than urban counterparts. The odds for moderate/severe anaemia among adolescent boys were relatively higher among late adolescents, with no mass-media exposure, stunted, and rural adolescents. Similarly, odds for moderate/severe anaemia among adolescent girls were higher among late adolescents and adolescents without schooling and mass-media exposure. Prevalence of anaemia was higher among adolescent girls than in boys. Lower education status, rural residence, late adolescence, no exposure to mass media, and stunting were the predictors of moderate/severe anaemia among adolescents. Anaemia among adolescents must be addressed through effective public health policy targeting adolescents residing in rural areas. There is a need to disseminate information about anaemia-related programs, such as National Iron Plus Initiative (NIPI), through mass media, and subsequently, the public health system may be prepared to tailor the needs of adolescent boys and girls.

## Introduction

Anaemia is a public health concern affecting both developed and developing countries with significant consequences for both human health as well as social and economic development^1^. It is a condition in which the number and size of red blood cells or haemoglobin concentration are lower than the established cut-off value, consequently impairing the blood's capacity to transport oxygen around the body^2^. Globally, anaemia affected 1.93 billion people in 2013, collectively causing 61.5 million years of life lived with disability (YLDs)^3^, and the prevalence was highest in Africa and South-East Asia region^1^. According to WHO guidelines for controlling iron deficiency anaemia, nutritional anaemia is a major public health concern in India, primarily due to iron deficiency^4^. A population-based study shows that 28.4% of the 14,300 Indian adolescents were anaemic, and the major causes of anaemia were vitamin B12 deficiency (25.6%), iron deficiency (21.3%), dimorphic anaemia (18.2%), and anaemia of inflammation (3.4%)^5^. Moreover, small studies conducted among adolescents from different parts of India reported that the prevalence of anaemia was 61.5% in Gujarat, 52.5% in Madhya Pradesh, 41.1% in Karnataka, 50% in Bihar, and 56.3% in Uttar Pradesh^4,6–9^.

In adolescents, anaemia has been linked to affecting physical disorders, growth, and mental retardation, and also increases reproductive morbidities among adolescent girls during their womanhood^10^. Anaemia caused due to iron deficiency may reduce infection resistance, impaired physical growth and mental development, and reduced physical fitness, work capacity, and school performance^11^. Besides, when anaemic adolescent girls become pregnant, they are exposed not only to the risk of maternal morbidity and mortality but also the incidence of premature delivery, low birth weight, and perinatal mortality, and also infants born to anaemic mothers have a greater risk of anaemia in the first six months of life^12^.

Studies have also shown that socio-economic factors such as low economic status^13^, adolescent level of education^14^, housing condition, and hygiene practices^15^ influence the risk of anaemia. Media exposure can affect the anaemia level as exposure to mass media leads to increased awareness, which improves anaemia levels^16^. Studies are of the opinion that girls marrying during adolescence increase the risk of complications during pregnancy^17^. One such complication can be the higher level of anaemia among them^18^. A population-based study on anaemia among adolescent girls shows that as the household income increased from the lowest to the highest quartile, the prevalence of anaemia and iron deficiency anaemia decreases^19–21^. A study by Gebreyesus et al. found that adolescent girls in their early adolescence age were more likely to be anaemic than adolescents in their later age^22^. Further, Dey et al., in their study, found a significant association between women's level of education with anaemia^23^.

The background above justifies the adverse effects of anaemia on adolescents and warrants further study to explore the determinants of anaemia among adolescents. Previously several studies have examined prevalence and factors associated with anaemia; however, most of the available literature is limited to female adolescents, thus ignoring male adolescents^13,21–24^. Furthermore, most of the available literature was limited to community study with a limited sample size^13,25,26^. Most of the studies examined prevalence and factors associated with anaemia among adolescents aged 10–14 years or 15–19 years, thus missing out on a comprehensive analysis of adolescents aged 10–19 years^6,26^. Also, the existing studies on anaemia among adolescent boys and girls in Uttar Pradesh and Bihar are based on data from a small sample^4,9,27^, which may not represent the overall study population. Moreover, there are limited studies in Uttar Pradesh and Bihar regarding anaemia among adolescents using large sample data focusing on adolescent boys and girls. According to the Indian census 2011, the majority of the population in the states of Uttar Pradesh (i.e., 77.72%) and Bihar (88.70%) were living in the rural areas. Hence, this study tries to fill this gap to study the prevalence of anaemia and the associated factors among adolescent boys and girls residing in Uttar Pradesh and Bihar.

## Methods

### Data

Secondary data analysis was performed on cross-sectional survey data from the Understanding the Lives of Adolescents and Young Adults (UDAYA) project survey. The survey was conducted in two Indian states, namely, Uttar Pradesh and Bihar, in 2016 by the Population Council under the guidance of Ministry of Health and Family Welfare, Government of India. The UDAYA collected detailed information on family, media, community environment, assets acquired in adolescence, and transitions to young adulthood indicators. Uttar Pradesh and Bihar's sample size were around 10,350 (each) adolescents aged 10–19 years. Adolescents aged 10–19 years were eligible for the interview. The required sample for each sub-group of adolescents was determined at 920 younger boys (10–14 years), 2350 older boys (15–19 years), 630 younger girls (10–14 years), 3750 unmarried older girls (15–19 years), and 2700 married girls in both states. The UDAYA adopted a multi-stage systematic sampling design to provide the estimates for states as a whole as well as urban and rural areas of the states. A total of 150 primary sampling units (PSUs)—villages in rural areas and census wards in urban areas were visited in both states to conduct interviews in the required number of households. The 150 PSUs were further divided equally into rural and urban areas, that is, 75 for rural and 75 for urban respondents. The 2011 census list of villages and wards (each consisting of several census enumeration blocks [CEBs] of 100–200 households) served as the sampling frame for the selection of villages and wards in rural and urban areas respectively. This list was stratified using four variables: region, village/ward size, proportion of the population belonging to scheduled castes and tribes, and female literacy. The household sample in rural areas was selected in three stages, while in urban areas, it was selected in four stages. In rural areas, villages were first selected systematically from the stratified list described above, with selection probability proportional to size (PPS). In urban areas, 75 wards were first selected systematically with probability proportional to size, and within each ward, CEBs were then arranged by their administrative number, and one CEB was chosen randomly. Several CEBs adjacent to the selected CEB were merged to ensure at least 500 households for listing. The detailed information on the sampling procedure and survey design was published elsewhere^27^. This study's effective sample size was 20,594 adolescents (boys-5979 and girls-14625) aged 10–19 years in Uttar Pradesh and Bihar.

### Variable description

#### Outcome variable

Measurement of the hemoglobin levels of a representative sub-sample of adolescents using the HemoCue Hb 201+ analyzer was undertaken. This system uses a single drop of blood taken from a finger and drawn into a cuvette, and then inserted into a portable battery-operated instrument. The haemoglobin concentration is indicated on a digital read-out in less than one minute. Three levels of severity of anaemia were distinguished: mild anaemia (10–11.4 g/dl for 10–11-year-olds, 10–11.9 g/dl for 12–14-year-olds and non-pregnant girls in ages 15–19 years, 10–10.9 g/dl for pregnant girls in ages 15–19 years, and 12–12.9 g/dl for boys in ages 15–19 years); moderate anaemia (7.0–9.9 g/dl for 10–14-year-olds and girls in ages 15–19 years, regardless of pregnancy status at the time of the interview, and 9.0–11.9 g/dl for boys in ages 15–19 years); and severe anaemia (< 7.0 g/dl for 10–14-year-olds and girls in ages 15–19, regardless of pregnancy status, and < 9.0 g/dl for boys in ages 15–19 years)^27^. The anaemia was coded as 0 “no anaemia,” 1 “mild anaemia” and 2 “moderate and severe anaemia.” Moderate and severe anaemia category was combined due to the low sample size in the severe anaemia category. The analysis was further bifurcated into adolescent boys and girls as the data provide estimates separately for both categories.

#### Explanatory variables

Age was grouped into two categories, i.e., early adolescents (10–14 years) and late adolescents (15–19 years). This study provides data for two age groups (10–14 years and 15–19 years); therefore, we have categorized age into two categories. Education was recoded as no education, 1–7, 8–9, and 10 and above years of education. Working status was recoded as not working “no” and working “yes.” Working status was defined as the respondents who did paid work in the last one year. Media exposure was coded as no exposure, rare exposure, and frequent exposure. Media exposure was formed using whether the respondent was exposed to television, radio, or newspaper. Rare media exposure was defined as those exposed to media at least once a week/month and frequent media exposure consist of those exposed to media every day. Marital status was coded as “married” and “not married.” The question regarding marital status was only asked from adolescent girls aged 15–19 years. Received Iron folic acid (IFA) and deworming tablets were coded in no and yes. Body Mass Index (BMI) status was coded as “thin (BMI-for-age Z-score < -2SD)”, “normal (BMI-for-age Z-score ≥ -2SD and ≤ 1SD” and “overweight/obese (BMI-for-age Z-score >  + 1SD)”^27^. Stunting was coded as “stunted” and “not stunted.” Height -for-age Z-score of <  = -2SD was cut-for stunting among adolescents^28^. The wealth index was recoded as poorest, poorer, middle, richer, and richest. The survey measured household economic status using a wealth index composed of household asset data on ownership of selected durable goods, including means of transportation, and data on access to several amenities. The wealth index was constructed by allocating the following scores to a household's reported assets or amenities. Then the scores were divided into five quintiles. Caste was recoded as Scheduled caste and Scheduled tribe (SC/ST), and non-SC/ST. Religion was recoded as Hindu and non-Hindu. The category of non-Hindu was recoded because the frequency of other religions was very low; therefore, for the analytical purpose, the recoding was done in such a manner. Residence was available in data as urban and rural.

### Statistical analysis

Univariate and bivariate analyses were carried out to provide the unadjusted associations. The Chi-square test was used for bivariate analysis. The analysis was stratified by sex. Multinomial regression analysis was carried out to provide the adjusted estimates for the analysis. Multinomial logistic regression is used to model nominal outcome variables, in which the log odds of the outcomes are modeled as a linear combination of the predictor variables. The ratio of the probability of choosing one outcome category (e.g., mild anaemia) over the probability of choosing the baseline category (e.g., no anaemia) is often referred to as relative risk (and it is also sometimes referred to as odds, as we have just used to describe the regression parameters above). Relative risk can be obtained by exponentiation the linear equations yielding regression coefficients that are relative risk ratios for a unit change in the predictor variable. The coefficients were reported at a 95% confidence interval (CI). STATA 14 was used to perform the analysis of the present paper. All the methods were performed in accordance with the relevant guidelines and regulations.

### Ethics approval and consent to participate

The data is freely available in public domain and survey agencies that conducted the field survey for the data collection have collected a prior consent from the respondent. The data can be accessed from: https://dataverse.harvard.edu/dataset.xhtml?persistentId=doi:10.7910/DVN/RRXQNT.

## Results:

The sample distribution of the study population is presented in Table 1. A higher proportion of adolescents were from the late adolescent group^15–19^; about one-fourth of adolescent boys and one-third of girls had 10 & above years of schooling, respectively. Moreover, the percentage of adolescents with no schooling was 3% for boys and 13% for girls. Nearly 27% of adolescent boys and 17% of girls were working. Three-fourths of adolescent boys and half of the girls had frequent media exposure, and 19% of adolescent boys and 13% of girls received IFA and deworming tablets. One-fourth of adolescent boys and 13% of girls were thin; moreover, 26% of adolescent boys and 39% of girls were stunted.

**Table 1:** Socio-demographic profile of adolescents aged 10–19 years.

Background characteristics	Boys		Girls	
	Sample	Percentage	Sample	Percentage
**Age (years)**				
Early adolescents (10–14)	2084	34.9	1653	11.3
Late adolescents (15–19)	3885	65.1	12,972	88.7
**Educational status (in years)**				
No schooling	190	3.2	1890	12.9
1–7	2497	41.8	3939	26.9
8–9	1754	29.4	4093	28.0
10 and above	1528	25.6	4703	32.2
**Working status**				
No	4377	73.3	12,179	83.3
Yes	1592	26.7	2446	16.7
**Media exposure**				
No exposure	335	5.6	2703	18.5
Rare	1078	18.1	4212	28.8
Frequent	4555	76.3	7710	52.7
**Marital status**				
Married	N.A	N.A	5206	35.6
Unmarried	N.A	N.A	9419	64.4
**Received IFA and Deworming tablets**				
No	4856	81.4	12,780	87.4
Yes	1113	18.7	1845	12.6
**BMI status**^**a**^				
Thin	837	25.8	561	13.1
Normal	2323	71.7	3585	83.4
Overweight/obese	81	2.5	150	3.5
**Stunting status**^**a**^				
Not stunted	2372	74.4	2668	60.7
Stunted	817	25.6	1725	39.3
**Wealth index**				
Poorest	704	11.8	1971	13.5
Poorer	1193	20.0	2735	18.7
Middle	1374	23.0	3188	21.8
Richer	1391	23.3	3577	24.5
Richest	1308	21.9	3154	21.6
**Caste**				
SC/ST	1605	26.9	3784	25.9
Non-SC/ST	4364	73.1	10,841	74.1
**Religion**				
Hindu	5024	84.2	11,540	78.9
Non-Hindu	945	15.8	3085	21.1
**Residence**				
Urban	1030	17.3	2356	16.1
Rural	4939	82.7	12,269	83.9
**States**				
Uttar Pradesh	4069	68.2	9855	67.4
Bihar	1900	31.8	4770	32.6
**Total**	5969	100.0	14,625	100.0

NA: Not Available; SC/ST: Scheduled Caste/Scheduled Tribe; ^a^sample is low because of anthropometric measures eligibility; IFA: Iron Folic Acid.

**Figure 1:**
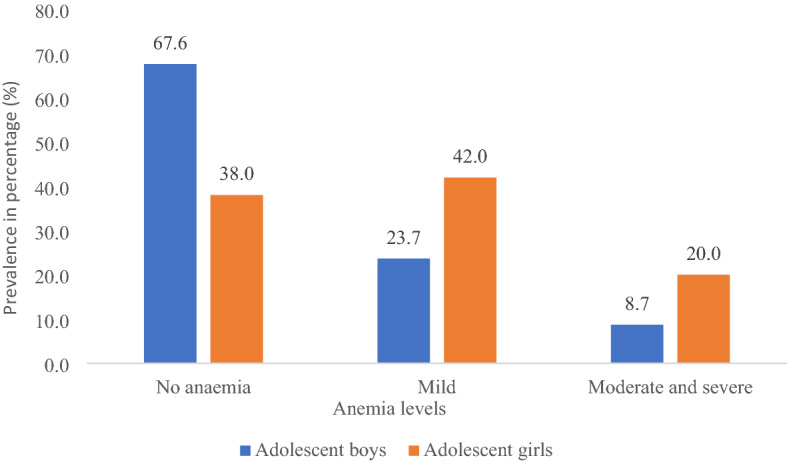
Prevalence of anaemia among adolescent boys and girls. Adolescent boys, adolescent girls.

Figure 1 displays the prevalence of anaemia among adolescent boys and girls aged 10–19 years. Overall, adolescent girls suffered more from mild (42% vs. 23.7%) and moderate/severe (20% vs. 8.7%) anaemia compared to adolescent boys.

Percentage distribution of anaemia among adolescent boys and girls by background characteristics is presented in Table 2. The prevalence of mild anaemia was significantly higher among early adolescents; however, moderate/severe anaemia was more prevalent among late adolescents, irrespective of their gender. The prevalence of moderate/severe anaemia was significantly higher among adolescent boys with no schooling, and it was substantially lower among adolescents who had 10 & above years of schooling. Similar results were found in the case of adolescent girls. A higher percentage of working adolescent boy suffered from moderate/severe anaemia than not working. Similarly, moderate/severe anaemia was more prevalent among working adolescent girls than non-working girls. Mild anaemia was more prevalent among unmarried adolescent girls, and moderate/severe anaemia was more prevalent among married adolescent girls. Adolescents who received IFA and deworming tablets suffered less from moderate/severe anaemia than those who did not receive, irrespective of their gender. Stunted adolescent boys suffered more from mild or moderate/severe anaemia than those who were not stunted. The prevalence of mild or moderate/severe anaemia was significantly higher among the poorest adolescents, and it was lowest among the richest ones, irrespective of their gender. Moreover, moderate/severe anaemia was more prevalent among adolescent boys who belonged to SC/ST, and rural adolescent boys suffered more from moderate/severe anaemia than urban counterparts. Similarly, adolescent girls who belonged to SC/ST caste suffered more from moderate/severe anaemia compared to their counterparts.

**Table 2:** Percentage distribution of anaemia among adolescents boys and girls by background characteristics.

Background characteristics	Boys (N = 3186 )			Girls (N = 4690)		
	Mild (%)	Moderate/Severe (%)	p-value	Mild (%)	Moderate/Severe (%)	p-value
**Age (years)**			0.000			0.000
Early adolescents (10–14)	27.1	6.5		42.6	12.6	
Late adolescents (15–19)	18.7	11.9		41.7	23.6	
**Educational status (in years)**			0.000			0.000
No schooling	16.8	11.7		39.3	28.7	
1–7	27.0	8.3		40.9	16.9	
8–9	20.9	11.1		44.4	21.1	
10 and above	16.5	6.1		42.7	19.3	
**Working status**			0.013			0.047
No	23.7	7.5		41.8	19.5	
Yes	23.4	13.2		42.9	23.4	
**Media exposure**			0.001			0.000
No exposure	28.4	13.1		44.1	26.1	
Rare	25.1	7.8		40.2	18.0	
Frequent	22.9	8.6		42.2	18.9	
**Marital status**						0.000
Married	N.A	N.A		38.8	26.2	
Unmarried	N.A	N.A		44.4	15.3	
**Received IFA and Deworming tablets**			0.050			0.007
No	23.7	9.5		40.8	20.7	
Yes	23.7	6.1		49.0	16.1	
**BMI status**^**a**^			0.000			0.245
Thin	27.8	7.4		43.6	12.3	
Normal	22.8	9.5		44.2	18.4	
Overweight/obese	4.4	0.5		38.8	24.7	
**Stunting status**^**a**^			0.000			0.321
Not stunted	21.7	7.9		41.6	19.8	
Stunted	29.5	11.2		42.4	20.2	
**Wealth index**			0.000			0.033
Poorest	33.1	8.5		42.8	20.2	
Poorer	25.1	8.7		43.7	20.5	
Middle	20.9	9.2		43.0	19.9	
Richer	22.9	9.2		41.3	18.3	
Richest	20.2	7.8		39.2	21.7	
**Caste**			0.005			0.000
SC/ST	28.0	9.4		39.8	23.7	
Non-SC/ST	22.1	8.4		42.8	18.6	
**Religion**			0.018			0.020
Hindu	24.8	9.2		42.6	20.3	
Non-Hindu	17.4	5.8		39.6	18.6	
**Residence**			0.000			0.309
Urban	19.3	5.9		43.5	19.9	
Rural	24.5	9.2		41.7	20.0	
**States**			0.000			0.000
Uttar Pradesh	25.2	10.5		39.6	21.7	
Bihar	20.6	5.1		46.3	17.0	

SC/ST: Scheduled Caste/Scheduled Tribe; NA: Not Available; ^a^sample is low because of anthropometric measures eligibility; IFA: Iron Folic Acid.

Adjusted relative risk ratios obtained from multinomial logistic regression identified potential risk factors associated with mild and moderate/severe anaemia among adolescent boys and girls are presented in Table 3. The likelihood of mild anaemia was 0.73 times (p < 0.05) less likely, and moderate/severe anaemia was 3.68 times (p < 0.01) more likely among late adolescent boys compared to early adolescent boys. Moderate/severe anaemia was 0.24 times less likely among adolescent boys who had 10 & above years of schooling than adolescents with no schooling (p < 0.01). Adolescent boys who had frequent media exposure were 0.49 times (p < 0.05) less likely to suffer from moderate/severe anaemia, than those who had no exposure. Overweight/obese adolescent boys were 0.24 (p < 0.01) times less likely to suffer from mild anaemia, than those with normal BMI. The likelihood of mild and moderate/severe anaemia was 1.51 and 1.64 times more likely among stunted adolescent boys than those not stunted (p < 0.01). Rural adolescent boys were 1.49 times (p < 0.05) more likely to suffer from moderate/severe anaemia, compared to urban counterparts.

**Table 3:** Relative risk ratios (RRRs) obtained from multinomial logistic regression of anaemia among adolescents by background characteristics, India.

Background characteristics	Boys (N = 3186 )		Girls (N = 4690)	
	Mild RRR (CI)	Moderate/Severe RRR (CI)	Mild RRR (CI)	Moderate/Severe RRR (CI)
**Age (years)**				
Early adolescents (10–14) **(Ref.)**				
Late adolescents (15–19)	0.73**(0.55–0.97)	3.68***(2.45–5.53)	1.32**(1.06–1.64)	1.87***(1.4–2.5)
**Educational status (in years)**				
No schooling **(Ref.)**				
1–7	0.81(0.46–1.42)	0.78(0.36–1.69)	0.97(0.74–1.27)	0.70**(0.51–0.98)
8–9	0.70(0.39–1.25)	0.55(0.25–1.22)	1.08(0.82–1.43)	0.71**(0.51–1)
10 and above	0.43**(0.23–0.82)	0.24***(0.1–0.56)	0.93(0.69–1.25)	0.49***(0.34–0.72)
**Working status**				
No **(Ref.)**				
Yes	0.92(0.7–1.21)	0.80(0.54–1.19)	0.96(0.76–1.21)	1.07(0.8–1.44)
**Media exposure**				
No exposure **(Ref.)**				
Rare	0.85(0.54–1.32)	0.56(0.29–1.09)	0.71***(0.56–0.89)	0.75(0.55–1.01)
Frequent	0.78(0.51–1.18)	0.49**(0.27–0.9)	0.69***(0.54–0.87)	0.75(0.55–1.03)
**Marital status**				
Married **(Ref.)**	N.A	N.A		
Unmarried	N.A	N.A	0.90(0.74–1.1)	0.84(0.66–1.08)
**Received IFA and Deworming tablets**				
No **(Ref.)**				
Yes	1.04(0.82–1.31)	0.90(0.59–1.37)	1.25**(1.02–1.52)	1.06(0.80–1.41)
**BMI status**^**a**^				
Thin	1.01(0.82–1.25)	1.08(0.77–1.52)	0.94(0.76–1.17)	0.80(0.59–1.07)
Normal **(Ref.)**				
Overweight/obese	0.24***(0.10–0.55)	0.29*(0.07–1.22)	0.92(0.63–1.32)	0.78(0.46–1.30)
**Stunting status**^**a**^				
Not stunted **(Ref.)**				
Stunted	1.51***(1.23–1.87)	1.64***(1.18–2.28)	0.87(0.75–1.01)	0.84(0.69–1.02)
**Wealth index**				
Poorest **(Ref.)**				
Poorer	0.69**(0.5–0.95)	0.71(0.41–1.22)	1.0(0.77–1.29)	1.0(0.71–1.41)
Middle	0.66**(0.48–0.91)	0.86(0.52–1.44)	1.05(0.81–1.35)	1.13(0.81–1.58)
Richer	0.86(0.62–1.19)	0.79(0.46–1.37)	0.97(0.74–1.26)	0.86(0.60–1.22)
Richest	0.73^(0.52–1.04)	0.80(0.44–1.43)	0.85(0.64–1.14)	1.06(0.72–1.56)
**Caste**				
SC/ST **(Ref.)**				
Non-SC/ST	0.92(0.74–1.14)	1.08(0.76–1.55)	1.03(0.86–1.23)	0.84(0.67–1.06)
**Religion**				
Hindu **(Ref.)**				
Non-Hindu	0.70**(0.54–0.92)	0.60**(0.38–0.95)	0.82**(0.68–0.99)	0.77**(0.6–1)
**Residence**				
Urban **(Ref.)**				
Rural	1.22(0.99–1.5)	1.49**(1.05–2.11)	0.77***(0.66–0.91)	0.85(0.68–1.06)
**States**				
Uttar Pradesh **(Ref.)**				
Bihar	0.67***(0.55–0.82)	0.45***(0.32–0.63)	1.11(0.95–1.29)	0.77**(0.63–0.94)

RRR = Relative risk ratio; CI = Confidence interval; Ref. Reference; ***p < 0.001; **p < 0.05; base category = ‘not anaemic’; SC/ST: Scheduled Caste/Scheduled Tribe;

NA: Not Available; ^a^sample is low because of anthropometric measures eligibility; IFA: Iron Folic Acid.

On the other hand, late-adolescent girls were 1.32 (p < 0.05) and 1.87 (p < 0.01) times more likely to suffer from mild and moderate/severe anaemia, respectively, than early adolescent girls. Moderate/severe anaemia was 0.49 times less likely among adolescent girls who had 10 & above years of schooling than adolescents with no schooling (p < 0.01). Moreover, rural adolescent girls were 0.77 times (p < 0.01) less likely to suffer from mild anaemia than urban girls.

## Discussion

This study aimed at examining the prevalence and associated factors of anaemia among adolescents aged 10–19 years in two states of India, namely Bihar and Uttar Pradesh, separately for male and female adolescents. A study examined anaemia among adolescents aged 10–18 years^16^. Minimal literature examining anaemia among male and female adolescents aged 10–19 years is available in public domain^10^. This study found that around one-fourth (23.7%) of the adolescent boys and 42% of the adolescent girls had mild anaemia. In contrast, around 9% of the adolescent boys and one-fifth (20%) adolescent girls were moderately and severely anaemic. Mahajan et al., in their study on adolescent boys and girls aged 14–18 years, noticed the prevalence of anaemia of around 45% among adolescent girls and 16% among adolescent boys^6^.

The current study found that anaemia was more prevalent among adolescent girls than in boys. A higher percentage of adolescent girls had moderate/severe anaemia than adolescent boys. This could be because of the onset of the menstrual cycle among girls, which prompts physiological blood loss^10,15^. Furthermore, moderate/severe anaemia among late adolescent girls was higher than in early adolescent girls. Girls in late adolescence may get married by the time and become pregnant, further affecting their anaemia status^17^.

The current study noted a lower odds of moderate/severe anaemia among adolescent girls and boys with higher education than those without schooling. Previous studies have also acknowledged the importance of education in reducing the risk of anaemia among adolescents^13^. Not only adolescent’s education but studies have also noted the importance of parent education in reducing anaemia among adolescents^18^. Educated adolescents are well-informed about their nutrition choices, which may be attributed to their improved anaemia levels. Education-based health belief model was effective in promoting knowledge, attitude, and behaviour in preventing anaemia^28^.

Wealth was negatively associated with the higher odds of anaemia among male adolescents. Higher wealth was associated with a reduced odd of anaemia. Previously available literature also noticed that increased household wealth reduces the chances of anaemia among adolescents^14,29^. Children belonging to rich households tend to have improved nutritional status, which may be attributed to better anaemia levels than their counterparts in poor households^30^. Adolescents from poor households consume less diversified diets with poor micronutrients, resulting in higher anaemia^29^. As stated above, boys tend to consume more nutritious food than girls; thus, male adolescents had a lower risk of anaemia than their counterparts. However, it is to be seen how household wealth disintegrates boy and girl for anaemia and to seek the answer, we suggest more such studies shall be undertaken. The prevalence of anaemia was higher among rural adolescents than their counterparts in urban areas. Previous studies also noted rural–urban differences in anaemia and found severe anaemia prevalence was higher among respondents in rural areas than in urban areas^24,25^. In rural areas, girls generally get married early and become pregnant during the late adolescent period, thus increasing the risk of anaemia^15^. Poor accessibility to healthcare services, poor awareness, high poverty, and illiteracy in rural areas may be attributed to the poor anaemia levels among children in rural areas^25^. Furthermore, poor nutritional status in rural areas may also be attributed to the higher anaemia among children^25^.

## Strengths and limitations

The data is cross-sectional. Therefore, the current study could not establish causality between predictors and anaemia status among adolescent boys and girls; however, the current study contributes to the limited available literature in this understudied population group. Furthermore, the data is not pan-India and is limited to only two socio-economic backward states of India; therefore, findings shall not be generalized to the country population. Data on pregnancy status, malaria, and other confounding factors were not taken in this study which could have affected the estimates. However, this is also one of the potential strengths of the current study. Data were collected from Uttar and Bihar, and these two states have the highest adolescent population in India; therefore, the current study is very important.

## Conclusion

The prevalence of anaemia among adolescent boys and girls in the study area is a public health concern. The current study noticed that the prevalence of anaemia was higher among female adolescents than in male adolescents. Furthermore, moderate/severe anaemia was higher among late adolescents, adolescents without schooling, adolescents without media exposure, and rural adolescents. Strategies to improve iron status among adolescent boys and girls might reduce anaemia. However, in the current study, the association between IFA tablets and anaemia among female adolescents was not on the expected lines. Anaemia among adolescents must be addressed through effective public health policy targeting adolescents residing in poor households and rural areas. There is a need to disseminate information about anaemia-related programs, such as National Iron Plus Initiative (NIPI), through mass media, and subsequently, the public health system may be prepared to tailor the needs of adolescent boys and girls.

## Data Availability

The study utilizes secondary source of data which is freely available in public domain through https://dataverse.harvard.edu/dataset.xhtml?persistentId=doi:10.7910/DVN/RRXQNT. The necessary ethical approval has been taken by the respective organizations involved in the data collection process.

## Abbreviations

BMI: Body mass index
IFA: Iron-folic acid
RRR: Relative risk ratio
SC: Scheduled caste
SD: Standard deviation
ST: Scheduled tribe
UDAYA: Understanding the lives of adolescents and young adults
